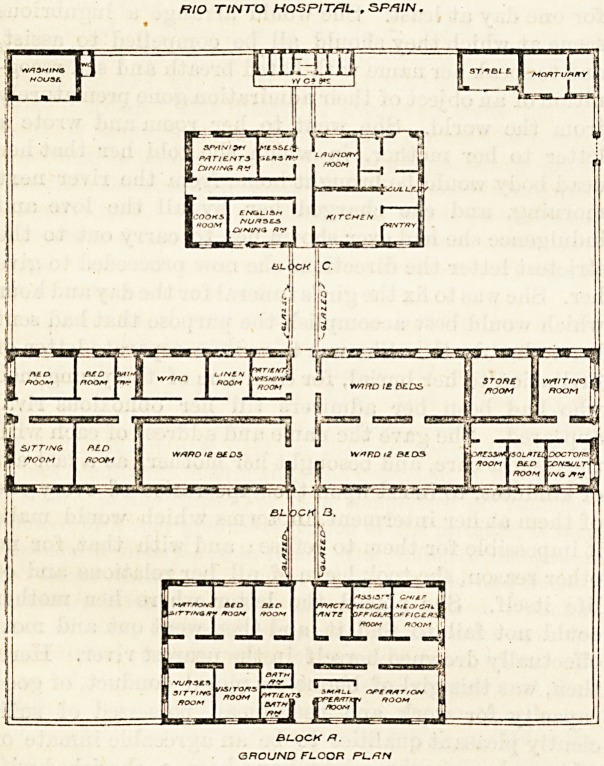# Hospital Construction

**Published:** 1899-09-09

**Authors:** 


### The Institutional Workshop.

### HOSPITAL CONSTRUCTION.

### THE RIO TINTO HOSPITAL, SPAIN.

The copper mines of Rio Tinto are said to be the largest of their kind in the world, and they provide occupation for about 9,000 people. Since the mines have been worked the ancient town of Onoba, the modern Huelva, has increased considerably in size and importance. It is about 50 miles from the mines, and is connected with them by a railway. The town of Rio Tinto is situated in a cup-like hollow among the hills, and the hospital is about 600 yards from the town.

The hospital is built of stone and roofed with tiles. It stands within an enclosure, the sides of which are formed partly by iron railings and partly by the walls of the building itself. It contains 44 beds. There are

RIO TINTO HOSPITAL, SPAIN.

BLOCK A.

GROUND FLOOR PLAN

three unequal rectangular blocks parallel to one another in their long axes, and about 25 ft. distant from each other. The blocks are connected by corridors having tiled roofs and glass sides, which are fitted up as conservatories, thus serving a double purpose. The vegetation in the neighbourhood is destroyed by the sulphur smoke from the mines, and the plants in these corridors cannot fail to be appreciated by the patients.

The floors are of cement, a system quite free from objection; in fact, decidedly advantageous in a climate like that of Spain. Hot and cold water are laid on, and there are electric bells throughout the building. It is proposed to substitute the electric light for the petroleum lamps at present in use.

Five rooms and a bath-room are set apart for the nurses, and the matron and the English nurse have each a large, well-furnished sitting-room and a bedroom leading from it. Another room is at their disposal for visitors.

Although the general design of this hospital may be commended, the carrying out of the details is open to much adverse criticism. Block "A" may, perhaps, be passed, as it is chiefly concerned with administration, and, presumably, contains such accommodation as may be required by the staff; but we think the position of the larger operating-room is faulty. There does not seem to be any good reason for placing it so near the entrance, and so far from the wards.

But it is in Block "B" that so many mistakes have been made. This block contains three dormitories for twelve beds each, and four small bedrooms. There are also a sitting-room and a bath-room. The extreme left-hand end of the block is taken up by these, and effectually shuts in three sides of the twelve-bedded ward which is placed between them and the corridor, leaving, therefore, only one side free to the open air. The opposite end of the block is similarly occupied by consulting-room, store-room, waiting-room, &c., and thus the ends of the large wards are as much enclosed as on the other side. Here, however, the two large dormitories are side by side; and, having doors in the divisional wall, are not so objectionable. Manifestly the smaller rooms ought to have been placed next the main corridor, and a cross corridor should have connected them, and then led on to the dormitories, which might have had either their long or their short diameters at right angles to the corridors, as best suited the climate and aspect.

There are no closets attached to this block. These are placed beyond the kitchen block, and, to reach them, patients must traverse the corridors and pass the kitchen on one hand and the nurses' sitting-room on the other.

It is curious to reflect that, in the construction of small hospitals, architects do not seem to study the plans of other hospitals. The Rio Tinto Hospital was rebuilt only seven years since, and yet, except its division into separate blocks, it hardly possesses a feature which authorities would nowadays insist on. The architect had only to turn up page 279 of "Burdett's Cottage Hospitals" to find a plan which, with very slight alterations, rendered necessary by object and climate, would have made an admirable hospital for Rio Tinto.

---

## Figures and Tables

**Figure f1:**